# OPN gene polymorphisms, *rs17524488* GG/G, *rs11730582* T/C, and *rs9138* C/A, and cancer risk in a Chinese population

**DOI:** 10.1038/srep14164

**Published:** 2015-09-15

**Authors:** Yuanyuan Mi, Kewei Ren, Feng Dai, Lijie Zhu, Ninghan Feng

**Affiliations:** 1 Department of Urology, Third Affiliated Hospital of Nantong University, Wuxi, 214000, P. R. China; 2 Department of Orthopedics, The Affiliated Jiangyin Hospital of Southeast University Medical School, Wuxi, 214000, P. R. China; 3 Translational Medicine Center, The Affiliated Wuxi Second People’s Hospital of of Nanjing Medical University, Wuxi 214000, P. R. China

## Abstract

Previous studies have investigated the association between osteopontin (*OPN*) gene polymorphisms, *rs17524488* (*−156 GG/G*), *rs11730582* (*−443 T/C*), and *rs9138* (*C/A*) and cancer risk in the Chinese population. However, the results are controversial and indefinite. We therefore carried out a meta-analysis to derive a more precise estimation of these associations. The PubMed database was systematically searched to identify potentially eligible reports. Crude odds ratios (OR) and 95% confidence intervals (CI) were used to assess the strength of associations between 3 OPN gene polymorphisms and cancer risk in a Chinese population. A total of 10 articles involving 2,391 cases and 3,007 controls were evaluated. The pooled OR indicated that *OPN rs17524488* (*−156 GG/G*) polymorphism was significantly associated with cancer risk in Chinese population. In a stratified analysis by source of control, significant associations were also observed among *rs17524488 (−156 GG/G*) and *rs11730582* (*−443 T/C*) polymorphisms and cancer. In addition, a stronger association was observed between *rs9138* (*C/A*) polymorphism and cancer risk. In conclusion, this meta-analysis suggests that *OPN rs17524488* (*−156 GG/G*), *rs11730582* (*−443 T/C*), and *rs9138* (*C/A*) polymorphisms may be associated with cancer susceptibility in the Chinese population. Nevertheless, further investigation on a larger population covering different ethnicities are warranted.

Cancer is a serious disease threatening public health worldwide. The estimates of newly diagnosed cancer cases/cancer-related deaths in worldwide and China were approximately 14.1/8.2 million in 2012 and 3.37/2.11 million in 2011, respectively^1^,^2^. The crude incidence was 235.23/10^5^ (268.65/10^5^ in males, 200.21/10^5^ in females)^3^ in China. Predisposition to cancer may be attributed to certain genetic polymorphisms that arise from single nucleotide polymorphisms (SNPs). In fact, numerous genome-wide studies of common cancers suggest a number of loci within the genome that, in spite of low-penetrance, may increase an individual’s susceptibility to cancer^4^,^5^,^6^.

Osteopontin (OPN) is a phosphorylated acidic glycoprotein with several functions in different physiological and pathological processes, including bone turnover, wound healing, ischemia, inflammation, autoimmune responses, and tumorigenesis, mediated by stimulation of certain signaling pathways via binding to cellular receptors, integrins, and CD44 variants^7^,^8^,^9^.

Overexpression of OPN has been described in several conditions in which basic inflammatory processes are activated, such as arthritis^10^, myocardial remodeling after infarction^11^, kidney interstitial fibrosis after obstructive uropathy and other renal insults^12^, wound healing^13^, and several types of cancer. This is because OPN is a metastasis-related gene that contributes to the progression of over 30 types of cancer^14^,^15^.

The gene encoding OPN, also known as secreted phosphoprotein 1 (SPP1), is mapped on human chromosome 4q21-q25, together with other members of the SIBLING family of proteins, bone sialoprotein, and dentin matrix protein-1, which share some structural characteristics^16^.

More than 10 SNPs have been identified in the OPN promoter. These polymorphisms may affect the transcriptional activity of OPN and some of them are thought to be genetic risk factors for disease susceptibility^17^,^18^,^19^. Several polymorphisms in the human gene encoding OPN have been identified in the Chinese population, of which the *rs17524488* (*−156 GG/G*), *rs11730582* (*−443 T/C*), and *rs9138* (*C/A*) polymorphisms are most frequently studied.

Considering the impact of the cancer risk potentially resulting from OPN gene, a number of studies have explored the association between these polymorphisms and cancer. However, individual studies have yielded inconsistent or conflicting findings, possibly caused by limitations associated with an individual study. To shed light on these contradictory results and to more precisely evaluate the relationship among OPN gene polymorphisms and cancer risk, we performed a meta-analysis of 10 published studies^20^,^21^,^22^,^23^,^24^,^25^,^26^,^27^,^28^,^29^, the original regions of which were all from China, no other ethnicities or regions existed.

## Methods

### Search strategy and inclusion criteria

We did our best to include all case–control studies published until date, regarding the association between *OPN rs17524488* (*−156 GG/G*), *rs11730582* (*−443 T/C*), and *rs9138* (*C/A*) polymorphisms and cancer risk. Eligible studies were found by searching the PubMed database for relevant reports published between 2010 and 2014. The search terms were “Osteopontin”, “polymorphism”, and “cancer”. In addition, the references of all retrieved articles were also manually searched for additionally relevant publications. The inclusion criteria were as follows: (1) evaluating the association between *OPN rs17524488* (*−156 GG/G*), *rs11730582* (*−443 T/C*), and *rs9138* (*C/A*) polymorphisms, and cancer risk in a Chinese population; (2) case–control study; (3) and sufficient information (*GG/GG, GG, GGG* for *rs17524488*; *TT, CC, TC* for *rs11730582*; and *CC, AA, CA* for *rs9138*) for calculating the pooled odds ratios (OR) with 95% confidence intervals (CI).

### Data extraction

Data included the following: first author, publication year, country, cancer type, source of control, each genotype frequency of the case and control groups, genotype methods, and the Hardy–Weinberg equilibrium (HWE) value in the control group.

### Statistical analysis

Odds ratios (OR) with 95% confidence intervals (CI) were assessed for determining the relationship between *OPN rs17524488* (*−156 GG/G*), *rs11730582* (*−443 T/C*), and *rs9138* (*C/A*) polymorphisms and cancer. The pooled OR was estimated for *rs17524488* (*−156 GG/G*) by homozygous (*GG vs. GG/GG*) and recessive models [*GG vs.*  (*GGG* + *GG/GG*)] as well as the allele model (*G vs. GG*); *rs11730582* (*−443 T/C*) by homozygous (*CC vs. TT*) and recessive models [*CC vs.* (*CT* + *TT*)] as well as the allele model (*C vs. T*); and *rs9138* (*C/A*) by homozygous (*AA vs. CC*), and recessive models [*AA vs.* (*AC* + *CC*)] as well as the allele model (*A vs. C*).

Heterogeneity was evaluated using a chi-square-based *Q*-test^30^, and the summary OR was determined with the *Z*-test. If *P* > 0.10 for the *Q*-test, a lack of heterogeneity among studies was found, meanwhile the fixed effects model should be used, otherwise, the random effects model should be used^31^,^32^. The HWE was assessed by a chi-square test in controls; *P* < 0.05 was considered significant. Sensitivity analysis was performed on excluded individual studies to assess the stability of the results. Publication bias was assessed by both Egger’s test and Begg’s test^33^. All statistical tests were used by Stata software (version 11.0; StataCorp LP, College Station, TX).

## Results

### Characteristics of Studies

**Figure 1:**
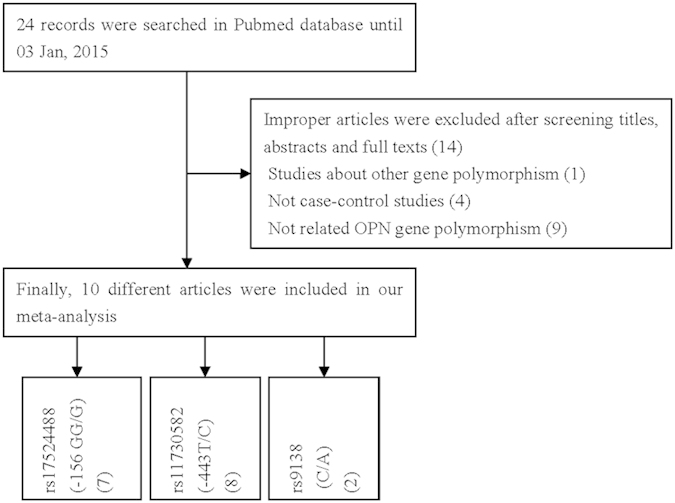
Flowchart illustrating the search strategy for OPN rs17524488 (−156GG/G), rs11730582 (−443 T/C) and rs9138 (C/A) polymorphisms and the risk of cancer.

Figure 1 and Tables 1, 2 show the study selection process and main characteristics of included studies, respectively. A total of 24 articles were retrieved based on the search criteria. Among them, 10 articles were excluded because they did not provide information about OPN gene polymorphism. An additional 4 articles, without control group data, were excluded. Thus, a total of 10 articles with 2,391 cases and 3,007 controls were included in the meta-analysis^20^,^21^,^22^,^23^,^24^,^25^,^26^,^27^,^28^,^29^. For the *rs17524488* (*−156 GG/G*) polymorphism, 7 studies were available, including a total of 2,130 cases and 2,644 controls. For the *rs11730582* (*−443 T/C*) polymorphism, 8 studies involved a total of 2,241 cases and 2,857 controls. For the *rs9138* (*C/A*) polymorphism, 2 studies involved a total of 418 cases and 424 controls. Among these, 2 studies focused on gastric cancer. The distribution of genotypes among controls was consistent with HWE in all but 2 studies^20^,^21^.

### Quantitative data synthesis

**Figure 2:**
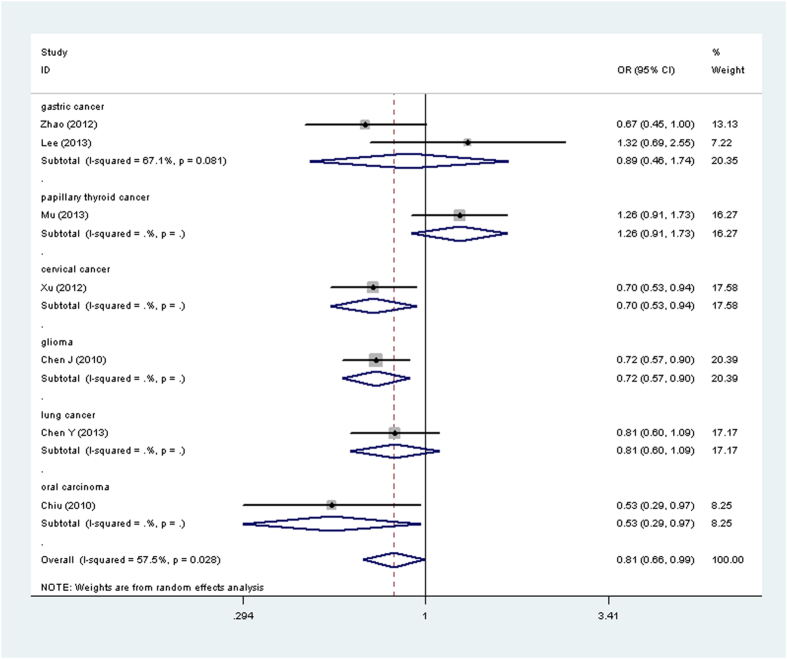
Forest plot of cancer risk associated with the OPN rs17524488 (−156 GG/G) polymorphism (GG vs. GGG+GG/GG) in the whole. The squares and horizontal lines correspond to the study-specific OR and 95% CI. The area of the squares reflects the weight (inverse of the variance). The diamond represents the summary OR and 95% CI.

**Figure 3:**
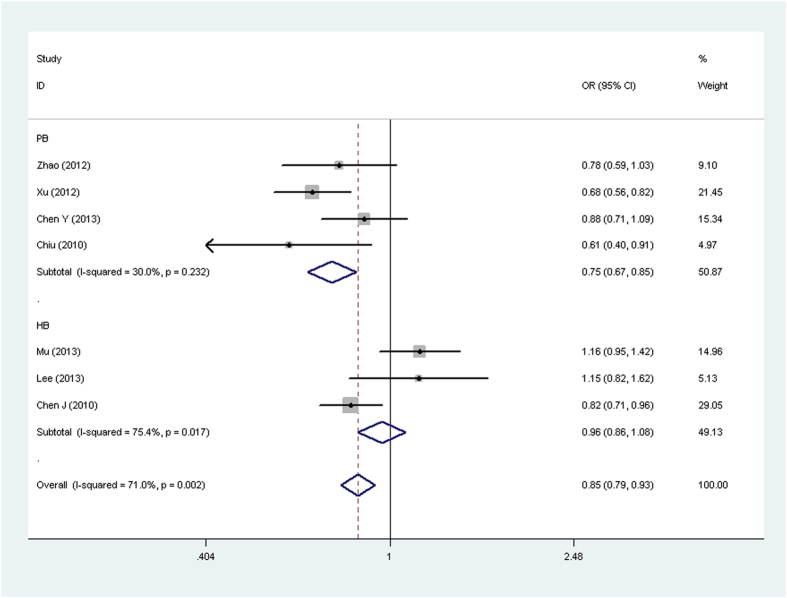
Forest plot of cancer risk associated with the OPN rs17524488 (−156 GG/G) polymorphism (G vs. GG) in the PB subgroup. The squares and horizontal lines correspond to the study-specific OR and 95% CI. The area of the squares reflects the weight (inverse of the variance). The diamond represents the summary OR and 95% CI.

**Figure 4:**
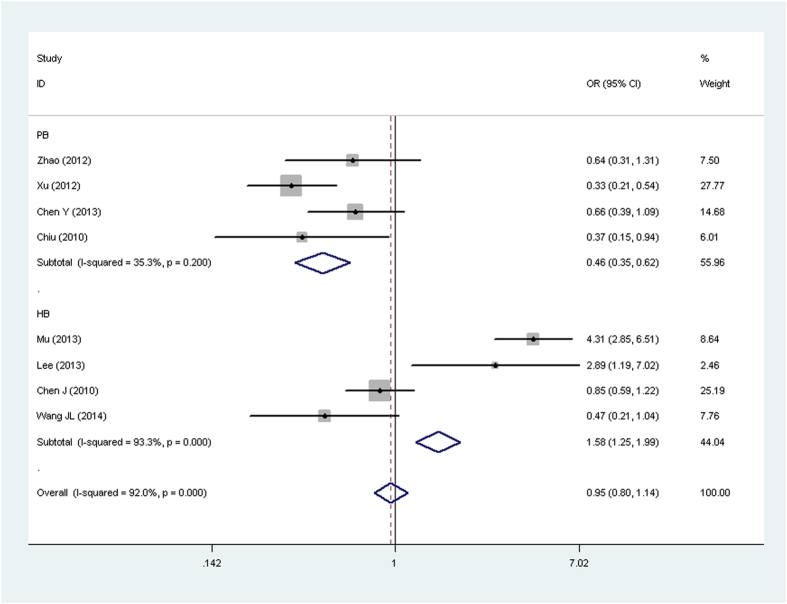
Forest plot of cancer risk associated with the OPN rs11730582 (−443 T/C) polymorphism (CC vs. TT) in the PB subgroup. The squares and horizontal linescorrespond to the study-specific OR and 95% CI. The area of the squares reflects the weight (inverse of the variance). The diamond represents the summary OR and 95% CI.

**Figure 5:**
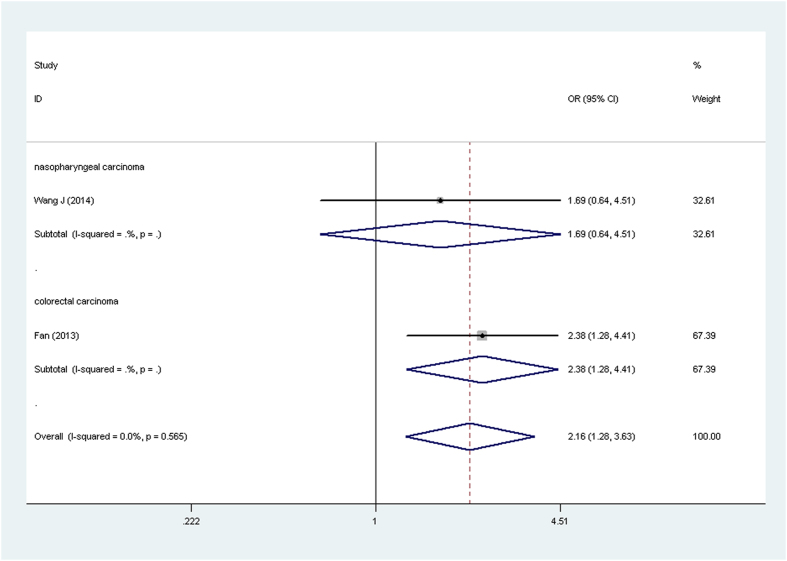
Forest plot of cancer risk associated with the OPN rs9138 (C/A) polymorphism (AA vs. CC) in the whole. The squares and horizontal lines correspond to the study-specific OR and 95% CI. The area of the squares reflects the weight (inverse of the variance). The diamond represents the summary OR and 95% CI.

Results of *OPN rs17524488* (*−156 GG/G*), *rs11730582* (*−443 T/C*), and *rs9138* (*C/A*) polymorphisms and cancer risk are presented in Table 3 and Figs 2, 3, 4, 5. For *rs17524488* (*−156 GG/G*) polymorphism, significant association was observed in all cancer-type combined studies (*GG vs. GGG* + *GG/GG*: OR = 0.81, 95% CI: 0.66–0.99, *P* = 0.028 for heterogeneity, *P* = 0.043). Subgroup analysis by source of control showed that statistically significant associations were present in PB (*GG vs. GGG* + *GG/GG*: OR = 0.81, 95% CI: 0.72–0.91, *P* = 0.558 for heterogeneity, *P* = 0.000; *GG vs. GG/GG*: OR = 0.84, 95% CI: 0.77–0.91, *P* = 0.103 for heterogeneity, *P* = 0.000; *G vs. GG*: OR = 0.75, 95% CI: 0.67−0.85, *P* = 0.232 for heterogeneity, *P* = 0.000). Two studies^20^,^21^ were not satisfied with the HWE, to make our analysis more powerful, we excluded these two studies and re-analysis. To our regret, no association was found, which indicated that the heterogeneity may exist in this *SNP*.

For the *rs11730582* (*−443 T/C*) polymorphism, significant association with cancer risk was observed in PB subgroup (*CC vs. CT* + *TT*: OR = 0.63, 95% CI: 0.49–0.82, *P* = 0.903 for heterogeneity, *P* = 0.000; *CC vs. TT*: OR = 0.46, 95% CI: 0.35–0.62, *P* = 0.200 for heterogeneity, *P* = 0.000). For the *rs9138* (*C/A*) polymorphism, significant relationship was detected overall (*AA vs. AC* + *CC*: OR = 1.62, 95% CI: 1.02–2.57, *P* = 0.883 for heterogeneity, *P* = 0.041; *AA vs. CC*: OR = 2.16, 95% CI: 1.28–3.63, *P* = 0.565 for heterogeneity, *P* = 0.004). To our regret, no association was found between gastric cancer and *OPN rs17524488* (*−156 GG/G*) or *rs11730582* (*−443 T/C*) polymorphism.

**Figure 6:**
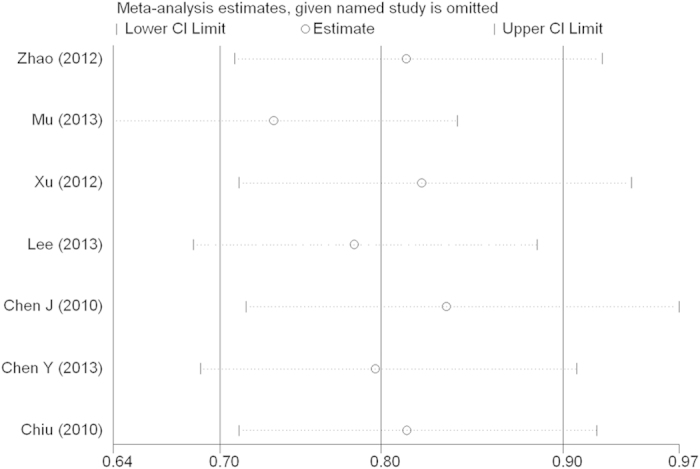
Sensitivity analysis between OPN rs17524488 (−156 GG/G) polymorphism and cancer risk.

**Figure 7:**
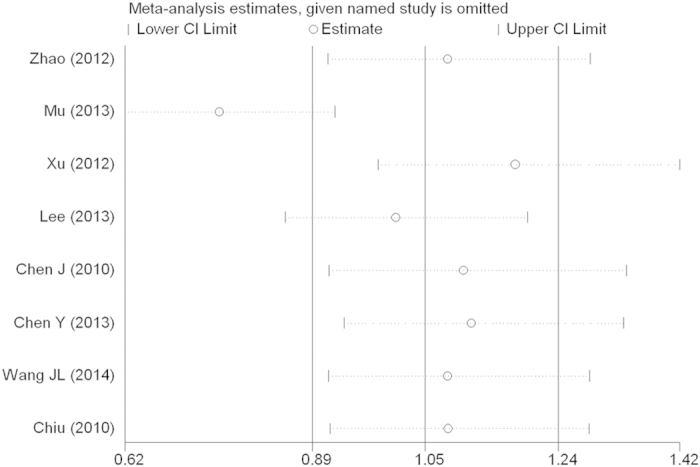
Sensitivity analysis between OPN rs11730582 (−443 T/C) polymorphism and cancer risk.

**Figure 8:**
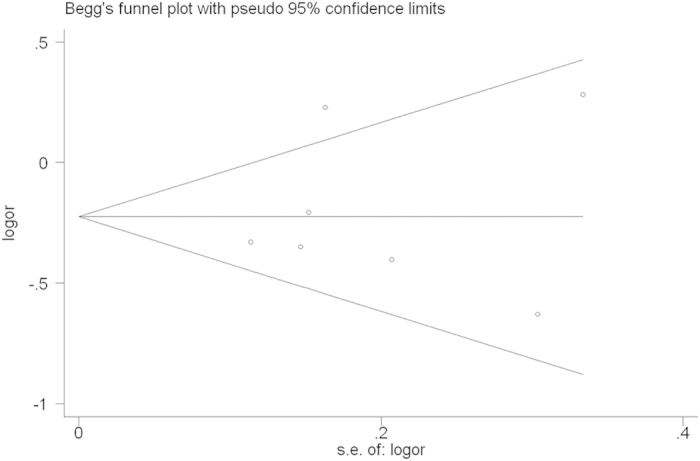
Begg’s funnel plot for publication bias test (GG vs. GGG+GG/GG in OPN rs17524488). Each point represents a separate study for the indicated association. Log [OR], natural logarithm of OR. Horizontal line, mean effect size.

**Figure 9:**
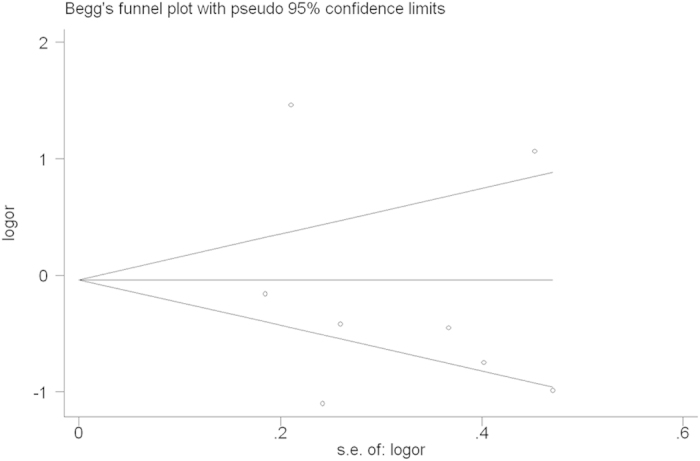
Begg’s funnel plot for publication bias test (CC vs. TT in OPN rs11730582). Each point represents a separate study for the indicated association. Log [OR], natural logarithm of OR. Horizontal line, mean effect size.

Sensitivity analysis and publication bias. Sensitivity analysis was used to determine whether modification of the inclusion criteria affected the final results. The sensitivity analysis did not influence the results excessively by omitting any single study for *rs17524488* (*−156 GG/G*) (Fig. 6). However, for *rs11730582* (*−443 T/C*), a single study named Mu *et al.*^23^ may influence the whole results (Fig. 7). Because only two studied of *rs9138* (*C/A*), the sensitivity analysis was not examed. Egger’s and Begg’s tests were performed to assess publication bias and the funnel plot symmetry was examined. Finally, no proof of publication bias was obtained (Table 4, Figs 8, 9).

## Discussion

The overall goal of a meta-analysis is to combine the results of previous studies to arrive at a summary conclusion about a body of research. It is most useful in summarizing prior research when individual studies are too small to yield a valid conclusion. In this study, we analyzed the associations between *OPN rs17524488* (*−156 GG/G*), *rs11730582* (*−443 T/C*), and *rs9138* (*C/A*) polymorphisms and cancer risk using a meta-analysis to obtain a powerful conclusion. To the best of our knowledge, this is the first meta-analysis providing comprehensive insights into the effects of the *OPN rs17524488* (*−156 GG/G*), *rs11730582* (*−443 T/C*), and *rs9138* (*C/A*) polymorphisms and risk associated with all types of cancer in a Chinese population. Our meta-analysis included 2,391 cases and 3,007 controls^20^,^21^,^22^,^23^,^24^,^25^,^26^,^27^,^28^,^29^.

For the *rs17524488* (*−156 GG/G*) polymorphism in the OPN promoter region, the overall results suggested that the subjects with G allele showed decreased susceptibility to cancer in a Chinese population. Moreover, individuals carrying either *−156 GG* or *−443 CC* genotype may have lower cancer susceptibility. However, people with *AA* genotype may have decreased cancer risk than *CC-*, *CT*, or *CC* + *CT* carriers. Considering that the previous single-institution study for cancer had a small sample size and may not justify the significance of current work, further studies are needed to clarify the effect of the 3 polymorphisms on the risk of cancer. A possible explanation for this phenomenon is that different polymorphisms may exert different effects on gene function, subsequently resulting in varying cancer susceptibility. Furthermore, a single gene or a single environmental factor is not likely to have a large effect on cancer susceptibility. Complex interactions between several genetic and environmental factors may be involved in cancer development.

Meta-analysis has been recognized as an effective method to answer a wide variety of clinical questions by summarizing and reviewing previously published, quantitative research. However, some limitations in our meta-analysis should be mentioned. First, our results were based on unadjusted estimates; more accurate outcomes would result from adjustments for other confounders such as gender, age, body mass index, lifestyle, and so on. Second, the studies included in this analysis were insufficient, especially in terms of a subgroup analysis. Thus, potential publication bias is very likely to exist, in spite of no evidence obtained from our statistical tests. Third, language of studies was limited to English, which may result in potential language bias. Fourth, a comparison of mRNA expression levels of the OPN gene between cancer and normal tissue should have been reported and included, which would better explain genetic function. Next, inter-gene and gene-environment interactions had not been evaluated owing to the absence of original data. Fifth, our study focused on Chinese people, other ethnicities should be reported and included. Sixth, a single study^23^ may influence the whole results in the sensitivity analysis for the *rs11730582* (*−443 T/C*) polymorphism, which suggested our study may be poorly powerful and stable. Finally, we also could not integrate different studies to look at the association between these three *OPN SNPs* and one specific cancer type, owning to insufficient publications. If there have a number of studies related different cancers in the future, this work may be carried out. In contrast, some advantages should also be highlighted. Our analysis comprehensively and systematically sheds light on the relationship between *OPN rs17524488* (*−156 GG/G*)*, rs11730582* (*−443 T/C*), and *rs9138* (*C/A*) polymorphisms and the susceptibility to cancer in the Chinese population. Additionally, due to the larger sample size, our meta-analysis increases the power and plausibility of our conclusion when compared with previous, individual studies. Finally, the studies included in this analysis were published between 2010 and 2014; thus, these studies are quite recent.

In summary, our meta-analysis suggests that *OPN rs17524488* (*−156 GG/G*)*, rs11730582* (*−443 T/C*), and *rs9138* (*C/A*) polymorphisms are associated with cancer risk in the Chinese population. Larger sample sizes of different ethnic populations are required to confirm our findings.

## Additional Information

**How to cite this article**: Mi, Y. *et al.* OPN gene polymorphisms, *rs17524488* GG/G, *rs11730582* T/C, and *rs9138* C/A, and cancer risk in a Chinese population. *Sci. Rep.* **5**, 14164; doi: 10.1038/srep14164 (2015).

## Figures and Tables

**Table 1:** Basic information for the included studies of the association between OPN gene polymorphism sites and cancer risk.

**First Author[Ref]**	**Year**	**Country/Region**	**Cancer type**	**Source of control**	**Cases**	**Controls**	***P*-value**	**Method**
**rs17524488(−156 GG/G)**							GG/GG vs. GG	
Zhao^20^	2012	China	gastric cancer	PB	200	200	0.18	GeneCore Bio Technologies
Mu^23^	2013	China	papillary thyroid cancer	HB	363	413	0.147	TaqMan
Xu^25^	2012	China	cervical cancer	PB	300	774	0.001	TaqMan
Lee^26^	2013	China-Taiwan	gastric cancer	HB	146	128	0.464	pyrosequencing
Chen J^29^	2010	China	glioma	HB	664	669	0.07	PCR–LDR
Chen Y^21^	2013	China	lung cancer	PB	360	360	0.218	GeneCore Bio Technologies
Chiu^28^	2010	China-Taiwan	oral carcinoma	PB	97	100	0.01	sequencing
**rs11730582(−443T/C)**							CC vs. TT	
Zhao^20^	2012	China	gastric cancer	PB	200	200	0.22	GeneCore Bio Technologies
Mu^23^	2013	China	papillary thyroid cancer	HB	363	413	< 0.001	TaqMan
Xu^25^	2012	China	cervical cancer	PB	300	774	<0.001	TaqMan
Lee^26^	2013	China-Taiwan	gastric cancer	HB	146	128	0.022	pyrosequencing
Chen J^29^	2010	China	glioma	HB	667	672	0.508	PCR–LDR
Chen Y^21^	2013	China	lung cancer	PB	360	360	0.068	GeneCore Bio Technologies
Wang JL^24^	2014	China	nasopharyngeal carcinoma	HB	108	210	0.062	PCR-RFLP
Chiu^28^	2010	China-Taiwan	oral carcinoma	PB	97	100	0.03	sequencing
**rs9138 (C/A)**							AA vs. CC	
Wang J^27^	2014	China	nasopharyngeal carcinoma	HB	150	150	> 0.05	SNaPshot SNP genotyping
Fan^22^	2013	China	colorectal carcinoma	HB	268	274	0.007	PCR-RFLP

Abbreviation: HB: hospital-based; PB: population-based; PCR-RFLP: polymerase chain reaction-restriction fragment length polymorphism; PCR: polymerase chain reaction-ligation detection reaction.

**Table 2:** Basic information for the included studies of the association between OPN gene polymorphism sites and cancer risk.

**First Author[Ref]**	**Each genotype frequency**								**Mean ± SD(Age range), year**	
	**Cases**			**Controls**					**Cases**	**Controls**
**rs17524488(−156 GG/G)**	GG	GGG	GG/GG	GG	GGG	GG/GG	HWE	G%		
Zhao^20^	67	92	41	86	78	36	0.018	0.625	56.29 ± 3.46(NA)	55.67 ± 4.21(NA)
Mu^23^	104	187	72	100	219	94	0.217	0.507	38.6 ± 2.1(NA)	38.4 ± 4.3(NA)
Xu^25^	88	129	83	287	359	128	0.381	0.603	54.6 ± 5.74(NA)	54.5 ± 2.61(NA)
Lee^26^	26	72	48	18	64	46	0.57	0.391	60.02 ± 13.91(27–90)	61.4 ± 8.46(37–87)
Chen J^29^	220	345	99	273	306	90	0.772	0.637	NA	NA
Chen Y^21^	137	150	73	155	136	69	0.000	0.619	57.2 ± NA(24–81)	56.3 ± NA(23–87)
Chiu^28^	27	52	18	42	49	9	0.318	0.665	NA	NA
**rs11730582(−443T/C)**	CC	CT	TT	CC	CT	TT		C%		
Zhao^20^	15	94	91	22	93	85	0.646	0.342	56.29 ± 3.46(NA)	55.67 ± 4.21(NA)
Mu^23^	119	171	73	62	187	164	0.469	0.376	38.6 ± 2.1(NA)	38.4 ± 4.3(NA)
Xu^25^	24	49	227	106	334	334	0.126	0.353	54.6 ± 5.74(NA)	54.5 ± 2.61(NA)
Lee^26^	21	66	59	8	55	65	0.416	0.277	60.02 ± 13.91(27–90)	61.4 ± 8.46(37–87)
Chen J^29^	69	299	299	77	311	284	0.557	0.346	NA	NA
Chen Y^21^	31	165	164	44	163	153	0.954	0.348	57.2 ± NA(24–81)	56.3 ± NA(23–87)
Wang JL^24^	10	38	60	30	95	85	0.678	0.369	48.2 ± 10.5(NA)	47.8 ± 11.2(NA)
Chiu^28^	9	41	47	17	50	33	0.793	0.420	NA	NA
**rs9138 (C/A)**	AA	AC	CC	AA	AC	CC		A%		
Wang J^27^	12	51	87	7	57	86	0.526	0.237	50 ± NA(16–92)	57 ± NA(25–84)
Fan^22^	31	138	99	20	102	152	0.614	0.259	58.2 ± 10.5(NA)	57.6 ± 4.4(NA)

Abbreviation: NA: not available; HWE: the Hardy–Weinberg equilibrium value in the control group.

**Table 3:** Total and stratified subgroup analysis for each OPN gene polymorphism site and cancer.

**Variables**	**N**^**a**^	**Cases/Controls**	**Allele model**	**Recessive model**	**Homozygous model**
			**OR(95% CI) *P*^b^ *P*^*c*^**	**OR(95% CI) *P*^b^ *P*^*c*^**	**OR(95% CI) *P*^b^ *P*^*c*^**
**rs17524488(−156 GG/G)**					
Total	7	2130/2644	0.85(0.72–1.01) 0.002 0.060	0.81(0.66–0.99) 0.028 0.043	0.76(0.55–1.06) 0.002 0.109
HWE	5	1570/2084	0.86(0.68–1.09)0.000 0.211	0.76(0.47–1.23)0.000 0.260	0.84(0.63–1.13)0.010 0.255
HB	3	1173/1210	1.01(0.78–1.31) 0.017 0.921	1.01(0.65–1.59) 0.009 0.949	1.06(0.67–1.70) 0.046 0.799
PB	4	957/1434	0.75(0.67–0.85) 0.232 0.000	0.81(0.72–0.91) 0.558 0.000	0.84(0.77–0.91) 0.103 0.000
gastric cancer	2	346/328	0.94(0.64–1.37) 0.085 0.733	0.89(0.46–1.74) 0.081 0.742	0.95(0.79–1.14) 0.178 0.591
**rs11730582(−443T/C)**					
Total	8	2241/2857	0.86(0.58–1.30) 0.000 0.477	0.92(0.55–1.53) 0.000 0.739	0.85(0.42–1.72) 0.000 0.653
HB	4	1284/1423	1.18(0.69–2.01) 0.000 0.543	1.40(0.66–2.99) 0.000 0.383	1.51(0.53–4.30) 0.000 0.445
PB	4	957/1434	0.63(0.39–1.01) 0.000 0.055	0.63(0.49–0.82) 0.903 0.000	0.46(0.35–0.62) 0.200 0.000
gastric cancer	2	346/328	1.14(0.65–1.99) 0.016 0.653	1.25(0.34–4.69) 0.016 0.736	1.33(0.30–5.84) 0.009 0.709
**rs9138 (C/A)**					
Total	2	418/424	1.38(0.88–2.16) 0.047 0.159	1.62(1.02–2.57) 0.883 0.041	2.16(1.28–3.63) 0.565 0.004

Annotation: ^a^Number of comparisons; ^b^*P* value of *Q*-test for heterogeneity test; ^c^*P*-value of *Z*-test for significant test.

**Table 4:** Publication bias tests (Begg’s test and Egger’s test) for two OPN gene polymorphism sites and cancer.

**Compared genotype model**	**Begg’s test**		**Egger’s test**	
	**z-value**	***P*-value**	**t-value**	***P*-value**
**rs17524488(−156 GG/G)**				
Allelic contrast	−0.15	0.881	−0.03	0.976
Homozygote comparison	0.15	0.881	0.09	0.935
Recessive genetic model	0.45	0.652	0.11	0.918
**rs11730582(−443T/C)**				
Allelic contrast	−0.74	0.458	−0.53	0.617
Homozygote comparison	−0.49	0.621	−0.45	0.670
Recessive genetic model	−0.49	0.621	−0.83	0.439

Annotation: *P*-value of *Z*-test for significant test.
